# The impact of COVID-19 in medical practice. A review focused on Urology

**DOI:** 10.1590/S1677-5538.IBJU.2020.99.08

**Published:** 2021-02-03

**Authors:** Eduardo Mazzucchi, Fabio C. M. Torricelli, Fabio C. Vicentini, Giovanni S. Marchini, Alexandre Danilovic, Carlos A. Batagello, Miguel Srougi, William C. Nahas

**Affiliations:** 1 Universidade de São Paulo Faculdade de Medicina Hospital das Clínicas São Paulo SP Brasil Setor de Endourologia, Divisão de Urologia, Hospital das Clínicas da Faculdade de Medicina da Universidade de São Paulo, SP, Brasil.

**Keywords:** COVID-19 diagnostic testing [Supplementary Concept], Urology, Practice Management, Medical, Review [Publication Type], spike protein, SARS-CoV-2 [Supplementary Concept]

## Abstract

COVID-19 pandemic is a rapidly spreading virus that is changing the World and the way doctors are practicing medicine. The huge number of patients searching for medical care and needing intensive care beds led the health care system to a burnout status especially in places where the care system was already overloaded. In this setting, and also due to the absence of a specific treatment for the disease, health authorities had to opt for recommending or imposing social distancing to relieve the health system and reduce deaths. All other medical specialties nondirectly related to the treatment of COVID-19 had to interrupt or strongly reduce their activities in order to give room to seriously ill patients, since no one knows so far the real extent of the virus damage on human body and the consequences of doing non deferrable procedures in this pandemic era.

Despite not been a urological disease, the urologist needs to be updated on how to deal with these patients and how to take care of himself and of the medical team he works with.

The aim of this article is to review briefly some practical aspects of COVID-19 and its implications in the urological practice in our country.

## INTRODUCTION

COVID-19 is a rapidly spreading virus whose first manifestations as a viral pneumonia occurred in Wuhan, China, in December 2019 (1). The disease is caused by a beta coronavirus named severe acute respiratory syndrome coronavirus 2 (SARS-CoV-2) (1). Coronavirus is a family of enveloped, single-stranded RNA viruses that cause respiratory, enteric, hepatic, and neurologic diseases (2, 3). Human Coronavirus (CoV) infections are caused by α- and β-CoVs (2, 3). SARS coronavirus (SARS-CoV) and MERS coronavirus (MERS--CoV) are members of β-CoVs family (2) and share 79.5% and 50% of their genomes sequence with SARS-CoV-2, respectively (4, 5).

COVID-19 is highly contagious through SARS-CoV-2 virus-containing respiratory droplets of infected personnel resulting from coughing or sneezing. This is considered the main source of infection as they are propelled for approximately one to two meters and then they can be deposited on the oral, nasal or ocular mucous membranes of a nearby person. The virus can also be transmitted by human to human contact or still, contact with infected objects (6). The incubation period for the SARS-COV-2 ranges from 2-14 days (7). The infection by the SARS-CoV-2 can be asymptomatic or produce symptoms like fever (98%), fatigue (70%), dry cough (60%), anosmia and coryza. These symptoms can progress to dyspnea in 30% of the patients progressing as the pulmonary involvement increases (8). Eventually, non-respiratory symptoms like diarrhea and nausea occur in 10% of the cases (8). About 30% of patients will present an asymptomatic infection, 55% of patients will have mild to moderate symptoms and 15% severe to critical symptoms (7, 9). The mortality rate throughout the world and in Brazil is around 7% (10, 11). Older people and those with chronic co-morbidities (obesity, hypertension, diabetes) present with higher mortality rates (12).

Due to its rapidly spreading COVID-19 was defined as a pandemic by the World Health Organization in March 11^th^, 2020 (13). The disease is changing medical practice and the way of life throughout the World (14). By the end of April 2020, three million people have been affected with more than 200.000 people deceased (10). The sanitary and health systems were severely affected in the majority of countries reaching a burnout status in many of them. Surgical and Urological practice changed rapidly in the last months in order to adapt to the new sanitary conditions. The aim of this article is to evaluate the impact of COVID-19 in urological practice in a developing country and how to deal with it.

## MATERIALS AND METHODS

An extensive review of the existing literature on Pubmed was performed, including web pages of the World Health Organization (WHO), Center for Diseases Control (CDC) and Brazilian Council of Medicine (CFM). The protocols of the Brazilian Ministry of Health were also accessed. The terms searched were COVID-19; SARS-CoV-2; surgery; urology, operation, laparoscopy, pandemic, renal transplantation.

## RESULTS

Effects of SARS Cov-2 on the genitourinary tract SARS-CoV-2 has a spike protein that binds to the epithelial cells through the angiotensin-converting enzyme 2 (ACE2) receptors. Therefore, the human cells that express ACE 2, like the heart, esophagus, urinary bladder and kidney can be potential target cells for the SARS-COV-2 infection (5). Additionally, the liver, vascular system and testis can be affected by other mechanisms. Some patients with severe disease develop acute kidney injury (AKI), which requires continuous renal replacement therapy (CRRT) (1, 15).

### Effect of SARS- Cov2 on the kidneys

The exact mechanism of kidney involvement is unclear: postulated mechanisms include sepsis leading to cytokine storm syndrome or a direct cellular injury due to the virus (16). Histopathological studies of kidneys from six patients who died from COVID-19 and with impaired renal function revealed acute renal tubular damage (but not glomerular injury) and acute tubular necrosis in all cases (5, 15, 16). According to Li et al., 60% of patients had proteinuria and elevated levels of serum creatinine (SCr) were observed in 22% of patients, during treatment. Furthermore, 28% of patients gradually worsened and were diagnosed with acute kidney injury (AKI). Seven of 65 patients (10.7%) evaluated by the authors required dialysis. The mortality of these patients was 5.3-times higher than those without AKI (1, 15).

### Effect of SARS-Cov2 on the reproductive system

The blood-testis barrier does not protect against SARS-CoV-2 infection. As other viruses like mumps, HIV, hepatitis B and C, Epstein- Barr and papilloma can cause viral orchitis, also SARS-COVID 2 can lead to orchitis and even lead to male infertility (17). Histopathological studies from testis obtained from six patients who died of SARS-CoV-2 (not COVID-19) revealed spermatogenic cell apoptosis, germ cell destruction, few spermatozoa in the seminiferous epithelium, thickening of the basement membrane and leukocyte infiltration in all six specimens (7). A recent study showed that the testosterone to luteinizing hormone ratio in 81 patients with COVID-19 was dramatically decreased in comparison with 100 age-matched healthy counterparts (patients with COVID-19: 0.74; healthy men: 1.31, P <0.0001) (18).

Despite no clinical cases of orchitis related to COVID-19 have been described so far, a fertility evaluation of young men interested in having children after recovery from COVID-19 is advisable (1, 17).

### Impact on urologic practice Urological visits and outpatient procedures

It is highly recommended that patients suspect or positive for COVID-19 do not have any contact with the other patients. This way many hospitals have created special areas for treatment of such patients reducing the other activities only to non-deferrable procedures.

Routine deferrable appointments should be postponed for at least six months and electronic consultations should be performed when possible. Medical prescriptions should be delivered outside the hospital or preferably electronically once the Brazilian Council of Medicine and the Health Ministry authorized validated electronic prescriptions during the pandemic period (19).

Outpatients diagnostic and elective procedures should also be postponed especially those requiring general anesthesia or sedation. Ficarra et al. proposed deferring diagnostic urodynamic studies, cystoscopy for lower grade bladder tumors, replacement of ureteral stents and nephrostomy tubes, prostatic biopsies and intravesical therapy for low or intermediate grade bladder tumors for up to six months (20). On the other hand, the authors advise to not postpone cystoscopy and intravesical BCG for high grade vesical tumors and prostatic biopsies for suspected high tumors (20) (Table-1).

**Table 1:** Outpatient procedures during COVID-19 pandemic (Ficarra et al., modified).

Procedure	Indication	Consideration
Urodynamic studies	Postpone	
Prostatic biopsy	Postpone	Consider performing biopsy is suspected high grade tumor
Cystoscopy	Postpone	Exception: known or highly suspected high-grade tumor
Intravesical BCG or other agents	Postpone for low/intermediate grade tumor	Do not postpone for high grade tumor
Ureteral stent replacement	Postpone for up to six months	Evaluate case by case. Do not postpone patients with high risk of calcification
Extracorporeal shockwave lithotripsy	Postpone calyceal and other elective situations	Do not postpone: ureteral stones and calcified ureteral stents

### Preoperative care and general rules

**Figure 1:**
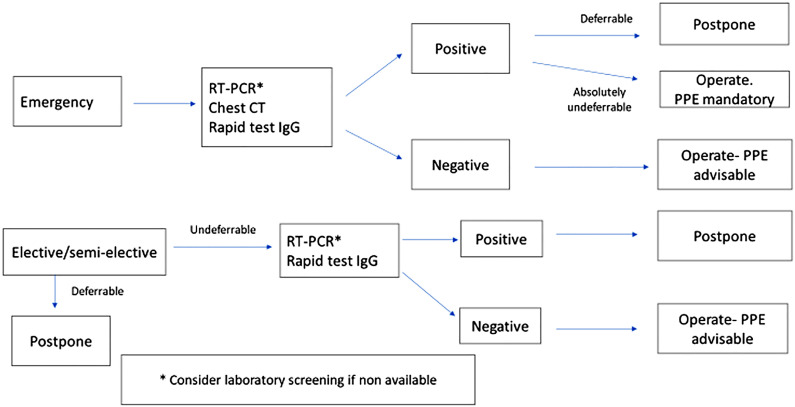
Preoperative flowchart during pandemic (Balibrea el at., modified).

Recent studies reported the occurrence of SARS-CoV 2 in urine, blood, anal swabs and stool and failed to demonstrate viral particles in the semen of patients with positive oro-pharyngeal swabs (21, 22) corroborating the data from Ling et al. (23) that reported the occurrence of SARS-Cov-2 RNA in the urine of 6.9% of patients recovering from COVID-19. Kumar et al. and others reported the blood isolation of SARS-CoV-2 in 15% of patients with COVID-19 (24, 25). These studies reinforce the importance of specific rules for protection of the operating room personal. If a non-deferrable procedure has to be performed, some rules must be followed:

1. Consent discussion with patients must cover the risk of COVID-19 exposure and the potential consequences;
2. It is highly recommendable to have a dedicated operating room (OR) with negative pressure and a separated access from the other ORs. Also, a separate anesthesia machine and experienced team of anesthesiologists and operating room nurses are important.
3. Testing asymptomatic patients for COVID-19 before surgery with the aim of protecting them and the surgical team is controversial, once the nasopharyngeal and oropharyngeal testing PCR-RT has a 60 to 70% sensitivity (26). The Center for Diseases Control recommends only testing for those presenting with symptoms (27). However, 5% to 80% of COVID-19 patients may be asymptomatic depending on the population studied (28). The Spanish Association of Surgeons and the Society of American Gastrointestinal and Endoscopic Surgeons (SAGES) recommend testing for all patients undergoing elective or urgent surgeries and there is a tendency towards testing all patients once test-kits become more disponible (personal view) (29, 30). Additionally, the surgeon must pay attention to the medications that patients are taking, once many of them receive hydroxychloroquine, which can be cardiotoxic, non-Gram-negative directed antibiotics and heparin, among others (29, 30). If the oropharyngeal swab PCR-RT for SARS-CoV-2 is not available and the patient is suspicious for asymptomatic infection, a clinical and laboratorial screening on symptoms (fever, dry cough, myalgia, anosmia) and on white blood cell count, D-dimer, hepatic enzymes and renal function tests and a chest CT should be performed. The rapid test for IgG/IgM should be also performed if available (29). The Spanish group proposed a flowchart for testing patients before surgery, shown in Figure-1 (29);
4. Only those considered essential staff should be participating in the surgical procedure and unless there is an emergency, there should be no exchange of room staff;
5. All members of the OR staff should use Protective Personal Equipment (PPE). Everyone in the operating-room (OR) should wear caps, personal protective glasses, N95 or PF2 mask, protective gowns for contacts, procedure gloves and shoe covers (31). The recommendation for using N 95 masks varies across the World with some recommending its use for all types of surgeries and others only in cases of COVID-19 confirmed patients or for surgery with potential for aerosolize the virus (32–35). Face shields should be used in high risk situations. These measures should be used in all surgical procedures during the pandemic regardless of known or suspected COVID-19 status. Placement and removal of PPE should be done according to control of infectious diseases CDC and other guidelines (29–31);
6. Surgical equipment used during procedures with COVID-19 positive or persons under investigation/suspected COVID-19 patients should be cleaned separately from other surgical equipment (Table-2).

**Table 2:** Urologic emergencies and treatment during COVID-19 pandemic. (Ficarra et al., modified).

Clinical condition	Treatment
Acute urinary retention	Urethral catheter or suprapubic tube under local anesthesia
Upper urinary tract obstruction	Ureteral stent or percutaneous nephrostomy under local or spinal anesthesia. Consider semirigid ureteroscopy for obstructing lower ureteral stones (spinal blockade).
Urinary retention – bladder clot	Evacuation by cystoscopy. Consider prostate or bladder tumor resection or fulguration
Spermatic cord torsion	Surgical orchidopexy. Consider manual distortion
Infection of prosthesis	Surgical removal of device
Priapism	Corpora cavernosa irrigation under local anesthesia Shunt under spinal blockade
Abscesses and Fournier Gangrene	Percutaneous drainage/debridement

### Inpatient urologic surgeries and emergencies

Generally, all patients with non-life threating pathologies should have their surgeries and other procedures re-evaluated and delayed. Lei et al. reported the results of 34 asymptomatic patients for COVID-19 operated in Wuhan for various types of surgeries ranging from cesarean and appendectomies to orthopedic and cancer surgeries and who developed COVID-19 in the postoperative period. All patients in this group developed pneumonia, 15 (44%) were recovered at ICU and seven (20.5%) died from COVID-19 related complications (36). These are scaring data and talk per se. Therefore, BPH treatments, reconstructive and prosthetic surgeries among others should be deferred.

Urologic emergencies must be treated without delay but avoiding invasive procedures is recommended. The main emergencies and treatments during COVID-19 pandemic are summarized in Table-3, according to Ficarra et al. (20).

**Table 3:** Oncological surgeries and procedures during COVID-19 pandemic (Ficarra et al., Stensland et al., modified).

Organ	Cancer condition	Surgical procedure
	Clinical T1a/b	Postpone. Consider ablative procedures
Kidney	Clinical T2	Partial/radical nephrectomy
	Clinical T3-T4	Radical nephrectomy/ thrombectomy in case of thrombus
	Small/ low grade	Postpone
Bladder	Noninvasive high-grade tumor > 2cm tumor at first diagnosis	Trans urethral resection
	Invasive Refractory CIS	Radical cystectomy + diversion
Prostate	High grade	Radical prostatectomy. Consider radiotherapy for selected cases
	Low/intermediate grade	Postpone. Radiotherapy?
Testis		Radical orchiectomy
	Low grade	Postpone.
Upper urinary tract	High grade, ≥T1c	Nephroureterectomy + lymph node dissection
Adrenal tumor	Smaller than 6 cm	Postpone
	Bigger than 6 cm	Adrenalectomy

### Oncology

Cancer patients are considered immunocompromised patients due to the nature of their disease and to the treatment they are submitted (chemotherapy, radiotherapy, or surgery). Due to more advanced age, the impossibility of receiving adequate medical care and to the fact that cancer patients have 3.5 folds risk of developing COVID-19 related serious events, all elective surgeries, chemotherapy and radiotherapy procedures in stable patients should be deferred (36–38). The decision of postponing cancer surgeries or not will also depend on patient condition/ decision and of the capacity of the hospital in providing intensive care units, ventilators and blood-bank among other resources needed for big cancer surgeries (20).

Cancer surgeries can be classified in four categories according to their urgencies (20, 38, 39).

1. Non-deferrable surgeries: procedures whose delay can jeopardize cancer-related outcomes:
  Renal cancer: radical or partial nephrectomy for clinical T2-4 tumors;
  Urothelial cancer: radical nephroureterectomy for high grade upper tract urothelial carcinoma;
  Bladder cancer: transurethral resection for high-risk non-muscle invasive bladder cancer, any high-grade bladder cancer, or tumors more than 2cm at the time of diagnosis. Radical cystectomy and urinary diversion for muscle-invasive bladder cancer or refractory carcinoma in situ;
  Prostate cancer: radical prostatectomy with pelvic lymph node dissection for high risk or locally advanced prostatic carcinoma; Cancer of the testis: radical orchiectomy; Adrenal cancer: adrenalectomy for tumor >6cm;
  Penile cancer: partial penectomy for clinical >T1G3 penile cancer.
2. Partial non-deferrable surgeries: are those that when postponed probably will not cause harm to patients:
  Renal cancer: partial or radical nephrectomy for clinical T1b tumors;
  Bladder cancer: Endoscopic resection for small low-grade bladder tumors;
  Prostate cancer: radical prostatectomy for intermediate or high-grade prostate cancer.
3. Deferrable surgeries: those that when postponed will cause minimal harm to patients:
  Renal cancer: partial nephrectomy for small clinical T1 b renal tumors;
  Bladder cancer: transurethral resection for small low-grade bladder tumors;
  Prostate cancer: radical prostatectomy for low/intermediate prostate cancer.
4. Replaceable surgeries
  Small low-grade renal tumors can be replaced by cryotherapy or radiofrequency ablation.
  These data are summarized in Table-4.

**Table 4:** Endourologic conditions and management during pandemic (Proietti et al., modified).

Condition	Management
Grade 1 - Emergencies (operate in no more than 24-48h)	-Obstructive pyelonephritis (any cause) - Ureteral stone: Obstructive anuria, refractory pain, worsening renal function. Obstruction in immunocompromised patients (tx, chemo). - Pre-stented patients with pain, infection, hematuria - Postoperative complications (fistula, abscess, avulsion, obstruction).
Grade 2- Urgencies (operate in no more than 2 weeks)	Obstructive ureteral stone - no pain; no spontaneous elimination after 4 weeks; Obstructive renal pelvic stone with nephrostomy
Grade 3 - Semi elective surgeries (operate in no more than 8-12 weeks)	Previous stent due to ureteral or renal stones; Renal stone with afebrile UTI or chronic renal function loss.
Grade 4- Elective surgeries	Asymptomatic non obstructive caliceal stones- non solitary kidney.

### Endourology

Renal colic and urinary stones management accounts for approximately 30% of the daily practice of the general urologist. Renal colic is one of the most frequent pathologies in the emergency room of every hospital. During pandemic, renal colic should be managed conservatively as much as possible (40). Adequate analgesia and medical expulsive therapy (MET) with alpha blockers or calcium channel blockers should be used according to the guidelines especially for ureteral stones between 5 and 10mm not accompanied by infection or massive hydronephrosis and with controlled pain. According to the literature, patients under MET showed superior spontaneous stone passage rates in patients with <10mm distal ureteral stones treated with α-blockers (77.3%) compared to placebo or no treatment (54.4%) (41, 42).

Patients should be kept home whenever possible avoiding admission to the ward and monitored for refractory pain and development of infection and sepsis. In the case of infection associated to an obstructive ureteral stone, decompression with a ureteral stent or a percutaneous nephrostomy or even with ureteroscopy and intracorporeal lithotripsy or stone removal with baskets in favorable cases should be performed promptly once these patients can progress to sepsis in up to 25% of the cases (41, 43). External strings for double J stents that can be removed at the office or even at home should be encouraged after uneventful procedures avoiding a hospital visit (44).

Percutaneous nephrolithotomy (PCNL) is generally a bigger procedure requiring general anesthesia and intensive care especially in older patients with co-morbidities like obesity, diabetes, hypertension or other cardiac diseases or in cases of big staghorn stones (44).

In order to spare ICU beds and respirators these procedures should be postponed except in very selected cases. In the pandemic setting Extracorporeal Shockwave Lithotripsy (SWL) appears as an interesting option once it can be performed ambulatory under local anesthesia or sedation. Both the American Urological Association/Endourological Society (AUA/ES) and European Association of Urology (EAU) guidelines recommend SWL as an effective and safe therapy for ureteral stones <10mm, once the median stone-free rates for SWL is 66.5% against 85% for ureteroscopy but the complication rate is lower for SWL (41, 42). For renal stones SWL also presents good results, especially for non-lower pole and <20mm stones with very low complication rates.

The peak of the pandemic is decreasing in several countries which does not mean that the World will get rid of the virus soon and that protective measures will be relaxed in the next months. Thus, changing some practice patterns and not simply postponing procedures are of utmost importance once stone patients suffer with pain and repeated infections. Therefore, SWL can be a very interesting alternative in this moment.

Proietti et al. proposed a classification for endourologic procedures regarding their urgency (40). We propose some changes adapting to our country. This classification is summarized in Table-5.

**Table 5:** Rules for laparoscopy and robotic surgeries during pandemic

Adequate incision to trocar diameter Use an exclusive 5mm trocar for insufflation/deflation Lower pneumoperitoneum pressure Reduce electrocautery power, use bipolar Deflate using the OR suction system or a filter Deflate only by the dedicated trocar Avoid sudden release of pneumoperitoneum

Grade 1 - Surgical emergencies: patients that should be operated in 24-48 hours: obstruction associated to infection, obstructive anuria in solitary anatomic or functional kidney or bilateral obstructive ureteral stones, obstructive ureteral stones in immunocompromised patients or in those with complications of previous endourologic procedures presenting with urinary fistula, abscess or obstruction.

Grade 2 - Urgencies: patients that should be operated in no more than two weeks, including obstructive ureteral stones with mild or no pain and obstructive renal pelvic stone with nephrostomy.

Grade 3 - Semi-elective surgeries: patients that should be operated in no more than 8 weeks: patients previously stented due to ureteral or renal stones or in cases of renal stones with afebrile UTI or chronic renal function loss.

Grade 4 - Elective surgeries: asymptomatic non-obstructive caliceal stones in non-solitary kidneys.

#### Laparoscopy and Robotics surgeries in the pandemic era

One of the characteristics of SARS-CoV-2 is its high infectivity and capacity to remain viable and infective for up to four hours in aerosols and days in stainless steel and plastic, similarly to SARS-CoV-1 (44). These facts raise concerns of infection of the operating room (OR) personnel during intubation/extubation and in laparos- copic/robotic surgeries. Spread of viruses through the smoke of electrocautery or laser devices during surgery is well documented for papillomavirus (HPV) and also for HIV (37, 45). The cautery smoke of 62% of plantar warts treated harbored HPV virus DNA (46–48). Although not proved for SARS-CoV-2, this should not be the exception and OR personnel should take extreme care to avoid contamination during these procedures (49).

In order to avoid or minimize the risk of contamination during laparoscopic and robotic procedures, some rules must be followed. The trocars should be adequate to the incision to avoid carbon dioxide (CO2) leakage; an exclusive 5mm trocar should be used for insufflation and deflation and a smoke evacuation filter used, the pneumoperitoneum should be reduced to the lowest possible as well as the electrocautery power, the pneumoperitoneum should be evacuated slowly and only by the exclusive trocar (20, 49, 50). Additionally, it is recommendable that also the console surgeon wears protective glasses and mask (50) (Table-6).

#### Renal transplantation

No evidence of transmission of SARS-CoV-2 by organ donation has been demonstrated but it has been shown the presence of the virus in the blood of 15% of patents with COVID-19; therefore, all solid organs are at risk of transmission (25, 51, 52).

Like other elective surgeries, renal transplants, particularly those with live donors should be deferred especially in places with a high incidence of COVID-19 (53). Renal transplants should be considered for highly sensitized patients (panel reactive antibodies - PRA >95%) or other urgencies like no access for dialysis. The transplantation team must check the availability of intensive care unit and ventilators and the capacity of the hospital of having these patients in COVID-19-free area and trained personnel for taking care of transplanted patients (51).

Regarding donors, it is recommendable and mandatory that all potential live and deceased donors be screened with PCR-RT nasal and oropharynx swab test and also with the rapid test. Additionally, a clinical and laboratorial screening should be performed regarding the previous occurrence of symptoms (fever, myalgia, dry cough, etc.) also including a chest CT (51). Living donor transplants should be postponed for 14 to 21 days if donors had been exposed to the virus, visited highly epidemic regions or presented COVID-19 related symptoms. The donor should be closely monitored during this period (51, 52).

Regarding the safety of the transplant surgical team, it is mandatory the use of PPE as discussed above for other surgeries. Organ procurement teams should avoid travelling to COVID-19 high incidence zones and work in parallel with other procurement teams in order avoid contact among them (51).

There are only few reports on the outcomes of transplanted patients that developed COVID-19; in one case, the patient recovered with initial cessation and subsequent reduction of immunosuppressive agents (54) and another case where the patient recovered without any reduction in immunosuppression (53). On that manner, there is no absolute consensus about how to deal with these patients.

## CONCLUSIONS

SARS-CoV-2 induces a renal tubular lesion compatible with acute tubular necrosis that can lead to acute renal insufficiency and a testicular lesion similar to other viral orchitis but with no clinical cases reported. Presential outpatient visits must be replaced by electronic consultations and the majority of invasive outpatient diagnostic and therapeutic procedures shall be postponed. Urgent surgeries in general urology, oncology and endourology are well defined and exceptions should be discussed case by case. Elective surgeries must be postponed. Patients undergoing undeferrable surgeries shall be screened for SARS-CoV-2 infection before their procedures and positive cases should have their surgeries postponed unless an organ or life-threatening emergency occurs. Medical team must protect themselves by wearing appropriate PPE and by adopting surgical techniques to reduce spreading of the virus. Kidney transplantation can be done in particular situations with very special concerns to the live donor, receptor and medical team health.
